# 1-FFT amino acids involved in high DP inulin accumulation in *Viguiera discolor*

**DOI:** 10.3389/fpls.2015.00616

**Published:** 2015-08-11

**Authors:** Emerik De Sadeleer, Rudy Vergauwen, Tom Struyf, Katrien Le Roy, Wim Van den Ende

**Affiliations:** Laboratory of Molecular Plant Biology, KU LeuvenLeuven, Belgium

**Keywords:** fructan:fructan 1-fructosyltransferase, inulin, degree of polymerization, *Viguiera discolor*, *Helianthus tuberosus*, site-directed mutagenesis

## Abstract

Fructans are important vacuolar reserve carbohydrates with drought, cold, ROS and general abiotic stress mediating properties. They occur in 15% of all flowering plants and are believed to display health benefits as a prebiotic and dietary fiber. Fructans are synthesized by specific fructosyltransferases and classified based on the linkage type between fructosyl units. Inulins, one of these fructan types with β(2-1) linkages, are elongated by fructan:fructan 1-fructosyltransferases (1-FFT) using a fructosyl unit from a donor inulin to elongate the acceptor inulin molecule. The sequence identity of the 1-FFT of *Viguiera discolor* (*Vd*) and *Helianthus tuberosus* (*Ht*) is 91% although these enzymes produce distinct fructans. The *Vd* 1-FFT produces high degree of polymerization (DP) inulins by preferring the elongation of long chain inulins, in contrast to the *Ht* 1-FFT which prefers small molecules (DP3 or 4) as acceptor. Since higher DP inulins have interesting properties for industrial, food and medical applications, we report here on the influence of two amino acids on the high DP inulin production capacity of the *Vd* 1-FFT. Introducing the M19F and H308T mutations in the active site of the *Vd* 1-FFT greatly reduces its capacity to produce high DP inulin molecules. Both amino acids can be considered important to this capacity, although the double mutation had a much higher impact than the single mutations.

## 1. Introduction

Fructans, water soluble polymers of fructose and an extension of sucrose, are the major reserve carbohydrates in the vacuole of about 15% of the flowering plant species (Hendry, 1993). Linear fructans include the β(2-1)-type fructans, inulin, with the smallest molecule being 1-kestotriose (older nomenclature: 1-kestose) and the β(2-6)-type, levan. Graminan-type fructans are branched and contain both β(2-1) and β(2-6) linkages. Neo-type fructans contain neokestose as backbone, and inulin- and levan neoseries can be discerned (Di Bartolomeo and Van den Ende, 2015). The type of fructan, the degree of polymerization (DP) and the amount of branching is entirely species- and tissue-specific (Van den Ende et al., 2000; Pavis et al., 2001; Vergauwen et al., 2003). Fructans are believed to protect the fructan accumulating plants against abiotic stresses (Hincha et al., 2007; Livingston et al., 2009), most probably by stabilizing membranes (Hincha et al., 2002; Vereyken et al., 2003; Van den Ende et al., 2005b; Livingston et al., 2009). Moreover, fructans may contribute to overall cellular reactive oxygen species (ROS) homeostasis by direct ROS scavenging mechanisms (Peshev et al., 2013; Peukert et al., 2014; Matros et al., 2015) and small fructans may act as phloem-mobile signaling compounds under stress (Van den Ende, 2013). Such mechanisms may be involved in so-called sweet immunity responses (Bolouri-Moghaddam and Van den Ende, 2013; Van den Ende and El-Esawe, 2014).

Inulin synthesis is the result of the combined action of sucrose:sucrose 1-fructosyltransferase (1-SST) and fructan:fructan 1-fructosyltransferase (1-FFT), where 1-SST catalyzes the formation of 1-kestotriose from two sucrose molecules, while releasing glucose. Secondly 1-FFT transfers fructosyl units from one inulin molecule to another to elongate the inulin chain. This elongation (and thus the specific characteristics of 1-FFT) greatly influence the fructan pattern in different species, relying on differences in acceptor substrate affinity between different 1-FFTs (Hellwege et al., 1998; Vergauwen et al., 2003). 1-FFTs producing high DP inulin molecules, as found in *Echinops ritro* (globe thistle), *Cynara scolymus* (globe artichoke), and *Viguiera discolor* (*Vd*), prefer (long) inulin chains as acceptor substrate, while low DP 1-FFTs, found in *Cichorium intybus* (chicory) and *Helianthus tuberosus* (*Ht*, Jerusalem artichoke), prefer 1-kestotriose or very short inulin molecules as acceptor substrate (Koops and Jonker, 1994; Hellwege et al., 1998). Both chicory and Jerusalem artichoke are used as a source for commercial inulin production (Kalyani Nair et al., 2010; Yang et al., 2015).

All plant fructosyltransferases (FTs), acid invertases and fructan exohydrolases (FEH) are members of the glycoside hydrolase family 32 (http://www.cazy.org/GH32.html) (Henrissat, 1991). So far only three plant crystal structures have been determined: 1-FEH IIa from *Cichorium intybus* (Verhaest et al., 2005), *Arabidopsis thaliana* cell wall invertase (Verhaest et al., 2006), and 6-SST/6-SFT from *Pachysandra terminalis* (Lammens et al., 2012) of which only the latter is a FT. These enzymes show a common fold namely a β-propeller, containing the active site and a β-sandwich domain. Although the β-sandwich domain may play a role in substrate specificity (Alvaro-Benito et al., 2010), it has been established that particular amino acids in the vicinity of the active site play a major role in substrate specificity (Ritsema et al., 2006; Altenbach et al., 2009; Van den Ende et al., 2009). Seven amino-acid motifs were identified to be particularly important in donor and acceptor substrate selectivity within the plant GH32 subfamily (Figure 4 in Lammens et al., 2012). Regarding acceptor substrate specificity in plant FTs, site-directed mutagenesis on vacuolar invertases led to the development of 1-SSTs (Ritsema et al., 2006; Schroeven et al., 2008). A similar approach was used to transform the 6^G^-FFT/1-FFT of *Lolium perenne*, an enzyme capable of synthesizing neoinulin and regular inulin series, into a 1-SST (Lasseur et al., 2009). All this structure-function research led to the definition of an evolutionary hotspot involved in acceptor substrate selectivity (Ritsema et al., 2006; Lammens et al., 2012).

*Viguiera discolor*, an Asteracean fructan accumulator from the Brazilian cerrado is known for the accumulation of its high DP inulin-type fructans linked to its high DP 1-FFT (Van den Ende et al., 2005a). It was suggested that the capacity to accumulate high DP inulin-type fructans may represent an adaptation to the special conditions of drought stress and burning (Van den Ende et al., 2005a), although the physiological advantage of accumulating higher DP inulin fructans in some Asteracean species remains enigmatic.

A more immediate reason for this study was the fact that the physicochemical properties and (biological) effects of higher DP inulin fructans are often more interesting, both for food and non-food applications, including medical applications (Waes et al., 1998; Van den Ende et al., 2005a; van de Wiele et al., 2007; Paßlack et al., 2012; Han et al., 2013; Vogt et al., 2013, 2014). Therefore, understanding the molecular differences between low DP and high DP 1-FFTs can open up possibilities to tailor those enzymes to our needs.

## 2. Materials and methods

### 2.1. Cloning and site-directed mutagenesis

**Figure 1:**
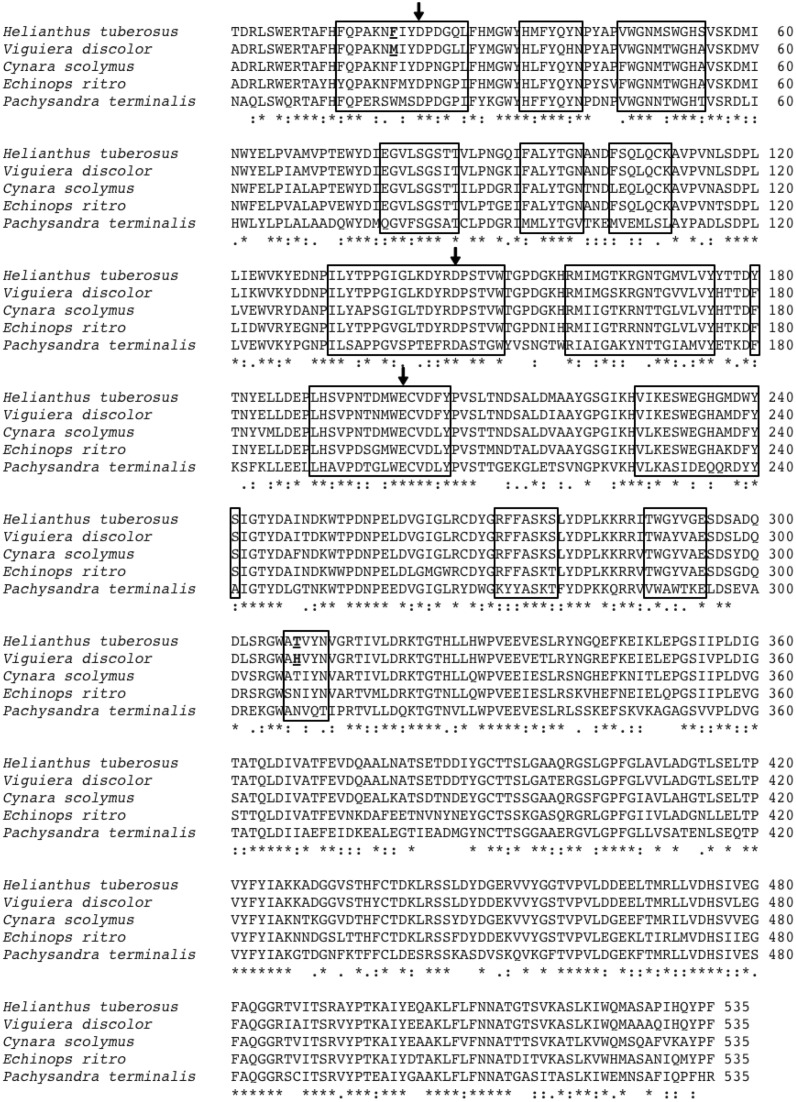
**Alignment of the amino acid sequences of the low DP 1-FFT of ***Ht*** and the three known high DP 1-FFTs of ***Echinops ritro***, ***Cynara scolymus*** and ***Vd*****. Also included is the sequence of the *Pachysandra terminalis* 6-SST/SFT on which the 3D homology models are based. The regions situated at a distance of 12Å or less from the catalytic triad (amino acids indicated with arrows) are indicated with a box. The amino acids, chosen for mutation, at positions 19 and 308 are underlined.

cDNA of the wild type (WT) 1-FFT from *Ht* (accession no. AJ009756, van der Meer et al., 1998) and *Vd* (accession no. AJ811625, Van den Ende et al., 2005a) was introduced in the *Pichia pastoris* expression vector pPICZαA. The expression vector containing the *Vd* WT 1-FFT cDNA was used as template to introduce a Met to Phe mutation in the KNMIY (M19F) and a His to Thr mutation in the WAHVY (H308T) region of this enzyme (Figure 1). Mutations were introduced through the QuikChange site-directed mutagenesis protocol (Agilent Technologies) with the following oligonucleotide primers (and their reverse complements): M19F: CAGCCTGCGAAGAATTTTATTTACGATCCAGATG and H308T: CTAGAGGATGGGCTACTGTTTATAATGTTG. After site-directed mutagenesis, the methylated template strand was digested by 1 μl of *DpnI* (37°C for 2 h) and purified by E.Z.N.A. Cycle Pure Kit (Omega bio-tek). Subsequently, 4 μl of the purified DNA was used to transform 40 μl of *E. coli* TOP10 cells through heat shock. Selection of positive colonies was done on low salt YT-zeocin (30 μg/ml) agar plates. The FastPlasmid Mini Kit (5Prime) was used to obtain and purify the plasmids from the positive colonies, after which sequencing (Macrogen, The Netherlands) was done to confirm the introduction of the desired mutations.

### 2.2. Heterologous expression in *Pichia Pastoris*

Expression plasmids containing the WT *Vd* 1-FFT, single mutants *Vd* M19F and *Vd* H308T and *Vd* M19F H308T double mutant along with the WT *Ht* 1-FFT were used to transform *Pichia pastoris*. Transformation and protein expression were performed as described in Schroeven et al. (2008) although methanol incubation was performed at 24°C.

Yeast supernatant, containing the recombinant enzymes was collected (1000 g, 4°C, 10 min) after 4 d of methanol incubation at 24°C. Sodium acetate buffer (200 μl, pH 5.0, 1 M) and (NH4)2SO4 (80% final saturation) were added before incubating on ice for 40 min. The pellet (40,000 g, 4°C, 20 min) was dissolved in 1 ml sodium acetate azide buffer [pH 5.0, 50 mM, 0.02% (w/v) sodium azide]. These enzyme solutions were centrifugated (15,000 g, 5 min, 20°C) to remove remaining insoluble impurities.

### 2.3. Enzymatic activity measurements

Enzymatic activity measurements were performed with 10 μl of dissolved pellet in 100 μl acetate buffer [pH 5.0, 50 mM, 0.02% (w/v) azide] with 100 mM 1-kestotriose as sole substrate, supplemented with 80 μM (final concentration in the reaction) of purified Burdock (*Arctium lappa*) fructo-oligosaccharides (BFO) with a DP of 9 or supplemented with 0.5% (w/v) of *Chicory* inulin (Sigma). Based on the mean DP of this inulin extract the final concentration is 500 μM (final concentration in the reaction). The reactions are executed on ice to prevent FEH activity, a side activity of these 1-FFTs that would complicate the interpretation of the data by hydrolyzing substrate and products in the reaction mixture. Samples of the reaction mixtures were taken [and diluted in azide mannitol water: 20 μM mannitol and 0.02% (w/v) sodium azide] at different time points and heated for 5 min at 90°C to stop the enzymatic reactions. Analysis of the reaction products was done by anion exchange chromatography (High-Performance Anion-Exchange Chromatography with Integrated Pulsed Amperometric Detection: HPAEC-IPAD, Thermo Fisher Scientific Dionex, Sunnyvale, CA, USA) as described by Van den Ende and Van Laere (1996). Peak areas were determined and compared to known amounts of standard compounds to quantify product formation.

After enzyme collection (see Section 2.2) test reactions were carried out to compare different activity levels. It was important for the succeeding reactions that the production speed of 1,1-kestotetraose and sucrose were similar for all enzymes (WT and mutants). To achieve this, all enzyme solutions were diluted accordingly with acetate buffer [pH 5.0, 50 mM, 0.02% (w/v) azide].

### 2.4. Burdock fructo-oligosaccharide purification

Burdock roots were lyophilized, crushed and extracted twice with water for 30 min at 90°C. After filtration impurities were removed from the extraction water by carbonatation, after a second filtration, charged molecules were removed by adding anion and cation exchange resin (Dowex H^+^ and Ac^−^). The solution was neutralized with sodium bicarbonate and lyophilized. The dry product was dissolved in 80% acetone to precipitate higher DP fructans (>DP15). The supernatant, containing all smaller fructans was subjected to rotary evaporation to remove the acetone and was lyophilized to increase the concentration of the fructans, ranging from DP3 to DP15. A Biogel P2 (Bio-Rad) was used to obtain a fraction of the fructan solution containing only inulins with a DP of 8, 9, or 10. After lyophilization to increase the concentration, this fraction was processed through preparative HPAEC to obtain a pure DP9 fraction. After neutralization with HCl the DP9 fraction was desalted on a Biogel P2 column.

### 2.5. 3D modeling

Homology modeling, based on the resolved 3D structure of 6-SST/6-SFT from *Pachysandra terminalis* (Lammens et al., 2012, sequence included in Figure 1) was done by MODELER (Sali and Blundell, 1993). Subsequently, 3D figures were created with PyMOL (Schrödinger, LLC, 2010).

## 3. Results and discussion

### 3.1. Alignment and modeling assisted mutant design

Although *Vd* 1-FFT and *Ht* 1-FFT share 91% of their amino acid sequence, both enzymes show a clear difference in enzymatic activity (Van den Ende et al., 2005a). *Vd* 1-FFT shows production of high DP inulin whereas *Ht* 1-FFT produces only short inulins.

3D homology models, based on the resolved structure of a 6-SST/6-SFT (Lammens et al., 2012) were used to make a primary selection of non conserved amino acids between the 1-FFT of *Vd* and *Ht* in the vicinity of the active site (residues at 12Å or closer to the catalytic triad, Figure 1 indicated with a box). Previous work in Altenbach et al. (2009) and Lammens et al. (2012) indicated several regions in the vicinity of the active site as important for acceptor binding specificity. More specific, Altenbach et al. (2009) suggested that N425 is an important amino acid in the acceptor substrate binding in *Schedonorus arundinaceus* 1-SST. A similar suggestion was made for N319 belonging to the GWA(N/S/T) motif in the *Pachysandra terminalis* 6-SST/SFT (Figure 4; Lammens et al., 2012). Both amino acids are equivalent with the H308 position in the Vd 1-FFT (Figure 1), making this a prime candidate for mutation.

**Figure 2:**
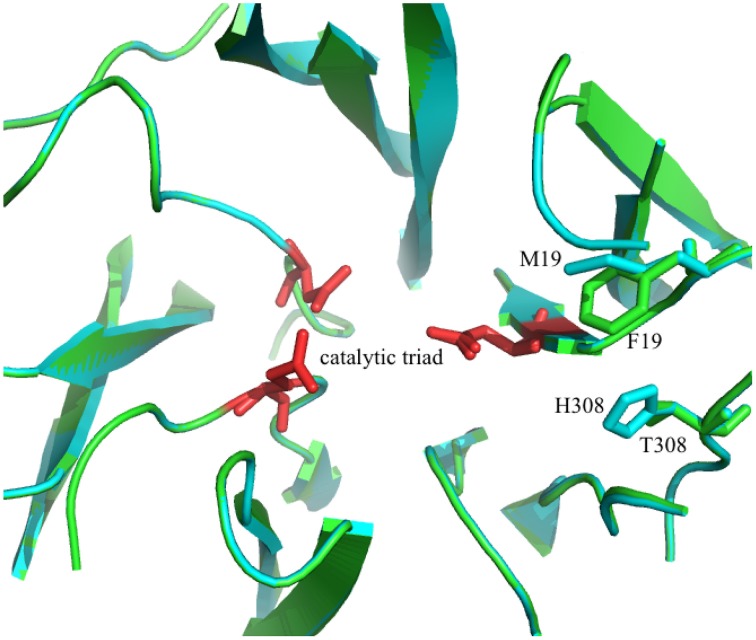
**3D model of the superimposed catalytic sites of the 1-FFT of ***Vd*** (blue) and ***Ht*** (green) (homology models, based on the 3D structure of 6-SST/6-SFT from ***Pachysandra terminalis*** Lammens et al., 2012)**. The amino acids of the catalytic triad are shown in red and the two mutations sites, M19F and H308T, are labeled.

A second candidate for mutation was found in M19, its location was suggested by Ritsema et al. (2006) and Lammens et al. (2012) to be involved with the binding of the acceptor molecule. Additional mutagenesis data from Schroeven et al. (2008) indicated a role for this position (W/Y23) in acceptor binding in wheat invertase/1-SST. Moreover M19 is located close to H308 (Figure 2), this is in line with the suggestion by Altenbach et al. (2009) that an acceptor substrate binding patch, consisting of several amino acids is formed at the rim of the active site, influencing the capability of the enzyme to use specific acceptor molecules.

### 3.2. Comparison of acceptor preference in *Vd* and *Ht* 1-FFT

A difficulty one needs to forestall while studying FFT activity is the potential for the products to be substrate in a subsequent reaction. Therefore, we use the most simple 1-FFT reaction possible, namely the reaction of two 1-kestotriose (GFF) molecules forming a sucrose (GF) and 1,1-kestotetraose (GFFF, old nomenclature: nystose) molecule (reaction 1).

(1) $$\text{GFF}+\text{GFF}\overset{1-FFT}{\to }\text{GF}+\text{GFFF}$$

The production of sucrose and 1,1-kestotetraose is followed over time to make sure all measurements are done while the production rate of sucrose and 1,1-kestotetraose is linear. While measuring during this linear phase and because of the excess 1-kestotriose we can be certain the only reaction occurring is (**1**). When incubation times are longer, the formed 1,1-kestotetraose can act as a fructosyl donor or acceptor, resulting in a non linear behavior of the 1,1-kestotetraose concentration. As an extra precaution, the formation of fructose (F) is monitored as this would be an indication of hydrolysis of 1-kestotriose and 1,1-kestotetraose molecules.

Plotting the concentration of sucrose and 1,1-kestotetraose produced in the reaction, in function of time (representative results are shown in Supplementary Figure 1) shows clearly the linearity during the first 4 h of this reaction for all WT and mutant enzymes. The *R*^2^-values of the linear trend line for all reactions are 0.95 or higher. The slope of each trend line is the rate at which sucrose and 1,1-kestotetraose are produced in each reaction.

**Figure 3:**
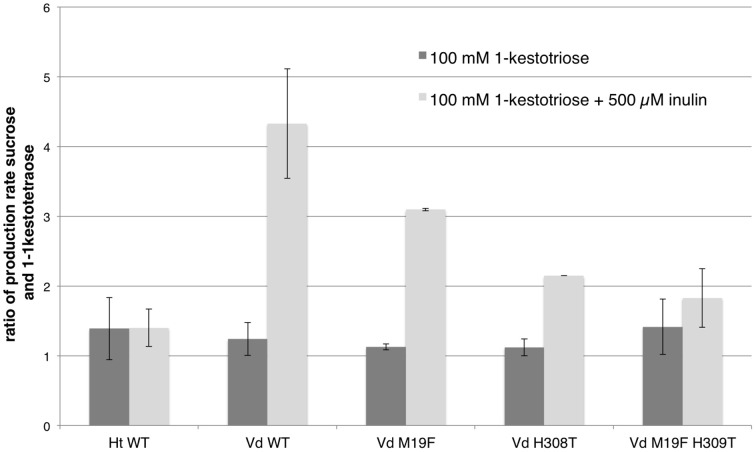
**Comparison of the mean (***n*** = 3, except for single mutants ***n*** = 2; with standard deviation indicated) of the ratios of the production rates for sucrose and 1,1-kestotetraose for both WT 1-FFTs, single mutant and double mutant ***Vd*** 1-FFT**. The dark gray bars show the ratio of production rates of sucrose and 1-kestotetraose when using 100 mM of 1-kestotriose as sole substrate. The light gray bars show the ratio when supplementing the reaction with 500 μM inulin.

The production rates for sucrose and 1,1-kestotetraose are very similar for each enzyme. This can be explained by the fact that the only enzymatic reaction taking place, is the reaction where two molecules of 1-kestotriose are converted in one sucrose and one 1,1-kestotetraose molecule (reaction 1). No other reaction products, indicating other (enzymatic) reactions, are detected in these samples. The stable fructose concentration (Supplementary Figure 1) shows that no fructans are being hydrolyzed. These similar production rates for sucrose and 1,1-kestotetraose result in a production rate ratio of around 1 (Figure 3, Supplementary Table 1).

When a similar setup is used to measure the enzyme activity with inulin supplementing 1-kestotriose as substrate, the enzymatic reactions of all enzymes (WT and mutants) are found to be linear as well (representative results are shown in Supplementary Figure 2) with *R*^2^-values of the linear trend line for all reactions being 0.95 or higher. Again this linearity is an indication that no reaction products are used as substrate in subsequent reactions.

Given the linearity of these reactions adding inulin to the reaction mixture gives rise to two additional reactions (other than reaction 1):

(2) $$\text{GFF}+{\text{GF(F)}}_{\text{n}}\overset{1-FFT}{\to }{\text{GF+GF(F)}}_{\text{n}+1}$$

and

(3) $$\text{GFF}+{\text{GF(F)}}_{\text{n}}\overset{1-FFT}{\to }{\text{GFF+GF(F)}}_{\text{n}-1}$$

Where reaction 1 gives rise to a production rate ratio of 1, because of the simultaneous production of sucrose and 1,1-kestotetraose, the occurrence of reactions 2 and 3 will influence this ratio since there is only production of sucrose or 1,1-kestotetraose. Reaction 2 will increase the ratio since sucrose is produced but no 1,1-kestotetraose is formed in that reaction. In a similar way reaction 3 will lower the ratio by producing 1,1-kestotetraose without sucrose. These ratios can be used to discriminate between the two additional reactions, this is important because it is impossible to measure the elongation or the shortening of the inulin molecules themselves due to the spreading of the total inulin peak area over a broad range of DP resulting in an abundant amount of very small peaks in the HPAEC-IPAD chromatograms.

Because of the excess 1-kestotriose in the reaction mixture (200:1), the reaction were one inulin molecule is elongated at the expense of another inulin molecule is not considered.

(4) $${\text{GF(F)}}_{\text{n}}+{\text{GF(F)}}_{\text{n}}\overset{1-FFT}{\to }{\text{GF(F)}}_{\text{n-1}}{\text{+GF(F)}}_{\text{n}+1}$$

When comparing the production rate ratios for both WT enzymes (Supplementary Table 1), it is apparent that the ratio for the incubations using 1-kestotriose as substrate are similar. Both enzymes using 1-kestotriose to produce sucrose and 1,1-kestotetraose at an equal rate, hence the ratios are close to 1. In contrast, the data from the incubations with supplemented inulin shows a clear difference. For the WT *Ht* 1-FFT a production rate ratio of almost 1 is observed for both setups (1-kestotriose and 1-kestotriose plus inulin) indicating that the 1-FFT of *Helianthus* is not using the inulin as a substrate (reactions 2 and 3) as this would result in an altered production rate ratio. On the other hand, when analyzing the data for WT *Vd* 1-FFT, a substantial difference between the ratio in the experiment with 1-kestotriose as sole substrate and the inulin supplemented experiment is revealed. In the latter case, the sucrose production is almost four times higher than the 1,1-kestotetraose production, indicating an increase in reaction 2 accounting for the increased sucrose production as compared to the 1,1-kestotetraose production.

In Figure 3 the production rate ratios of the different enzymes (WT and mutants) are plotted. When the production rate ratio of the inulin supplemented reaction of the WT *Vd* 1-FFT is compared to this of the double mutant it is clear that the ratio of this mutant is much lower than the ratio of the WT. This leads to the conclusion that this mutated enzyme has lost a great portion of the potential to perform reaction 2 compared to the WT. The single mutant 1-FFT enzymes show a lowered ratio that is situated between the values for the WT and the double mutant.

**Figure 4:**
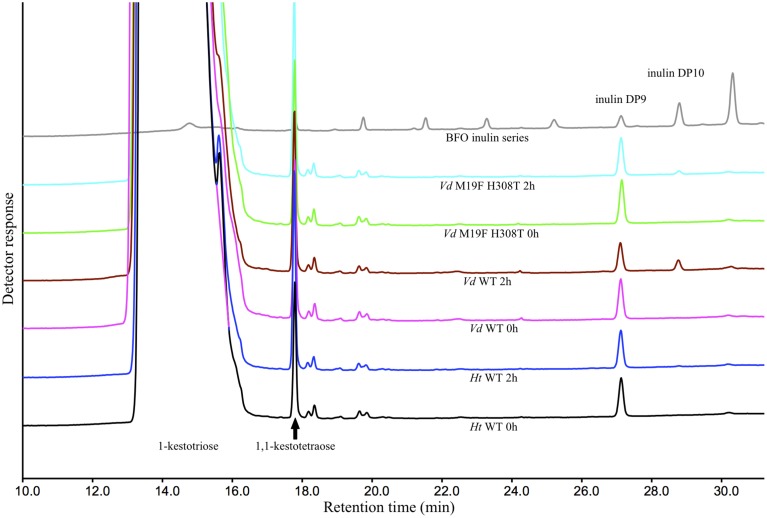
**HPAEC-IPAD chromatograms generated by the ***Ht*** and ***Vd*** WT and the M19F H308T double mutant at time 0 and after 2 h of incubation with 1-kestotriose (100 mM) and DP9 BFO (80 μ M) as substrate**. Also included is a chromatogram of a BFO series ranging from 1-kestotriose (DP3) to DP11.

These results could be confirmed when these reactions were repeated with the supplementation of purified DP9 BFO instead of inulin. After an incubation period of 2 h, a substantial amount of DP10 is, as expected, produced by the *Vd* WT 1-FFT. While in the reaction with the *Ht* WT 1-FFT, no production of DP10 is observed after 2 h. It is clear from the chromatogram of the *Vd* double mutant 1-FFT that the production of DP10 is drastically lowered compared to WT *Vd* 1-FFT, albeit not as low as the *Ht* WT 1-FFT (Figure 4).

The results from the inulin and DP9 BFO supplemented reactions indicate that the amino acids M19 and H308 play a major role in the acceptor substrate specificity of high DP inulins in the active site of *Vd* 1-FFT. While the capacity of the double mutant enzyme to use higher DP inulins as an acceptor is drastically reduced, it is clear that at least one additional amino acid should be found to bring this capacity at the level of the *Ht* 1-FFT.

Since only two mutations are introduced, and the activity of those mutants remained intact, it does not seem plausible that the introduced mutations an influenced the global fold of the proteins. It seems more likely that the mutations alter the local conformation of the acceptor substrate binding patch due to steric hindrance or the incompatibility of binding sites and as such influence the acceptor substrate binding.

The amino acids identified in this work are not conserved in the three high DP inulin producing 1-FFTs (Figure 1), suggesting that the acceptor substrate binding patch may be constructed in different ways. It appears that a specific combination of amino acids constructs a cavity in which long inulin chains can be captured for elongation. Destroying this cavity or disturbing the binding locations of specific acceptor molecules could limit the ability of the enzyme to use high DP inulins as acceptor substrate.

Based on the alignment data (Figure 1) and the results obtained in Lasseur et al. (2009) and Altenbach et al. (2009), the WEGHAMDFYS and WAYVAE regions may represent interesting locations to find additional amino acids influencing the acceptor substrate specificity of the *Vd* 1-FFT.

Finding the amino acids that are responsible for the different characteristics in each of these, structurally very similar, enzymes (and FTs in general) could provide us with the possibility of predicting the activity of FTs, solely on the sequence of the enzyme. An onset for this was already made in Lammens (2009) but much is left to be unraveled in this regard. Since more and more genomics data is available on an ever broadening range of species, predicting the activity could greatly facilitate research in this field. Being able to link certain enzymatic activities to certain amino acids in the active site could also give us the ability to construct enzymes that show the exact activity we need. This activity tailoring could prove useful when producing fructans with specific characteristics like the amount of branching or DP which could prove to be important in research on and the production of prebiotics and dietary fibers and fructan applications in general.

### Conflict of interest statement

The authors declare that the research was conducted in the absence of any commercial or financial relationships that could be construed as a potential conflict of interest.

## Abbreviations

1-FFT: fructan:fructan 1-fructosyltransferase
1-SST: sucrose:sucrose 1-fructosyltransferase
BFO: Burdock fructo-oligosaccharide
DP: degree of polymerization
FEH: fructan exohydrolase
FT: fructosyltransferase
HPAEC-IPAD: High-Performance Anion-Exchange Chromatography with Integrated Pulsed Amperometric Detection
*Ht*: *Helianthus tuberosus*
ROS: reactive oxygen species
*Vd*: *Viguiera discolor*
WT: wild type.
