# Supplementary material for: 1-FFT amino acids involved in high DP inulin accumulation in Viguiera discolor
Source: Front Plant Sci. 2015 Aug 11;6:616. doi: 10.3389/fpls.2015.00616 (PMC4531242; doi:10.3389/fpls.2015.00616)
Supplement: Supplementary file 1 [file Image1.PDF]

Supplementary Material: 1-FFT amino acids involved in high DP inulin accumulation in *Viguiera discolor*

Emerik De Sadeleer [1], Rudy Vergauwen [1], Tom Struyf [1], Katrien Le Roy [1] and Wim Van den Ende [1,*]

[1] *Laboratory of Molecular Plant Biology, KU Leuven, Leuven, Belgium*

Correspondence*:
Wim Van den Ende
Laboratory of Molecular Plant Biology, Institute of Botany and Microbiology, Kasteelpark Arenberg 31, B-3001 Leuven, Belgium,
Wim.VanDenEnde@bio.kuleuven.be

Fructans and RFOs in plants

1 SUPPLEMENTARY TABLES AND FIGURES

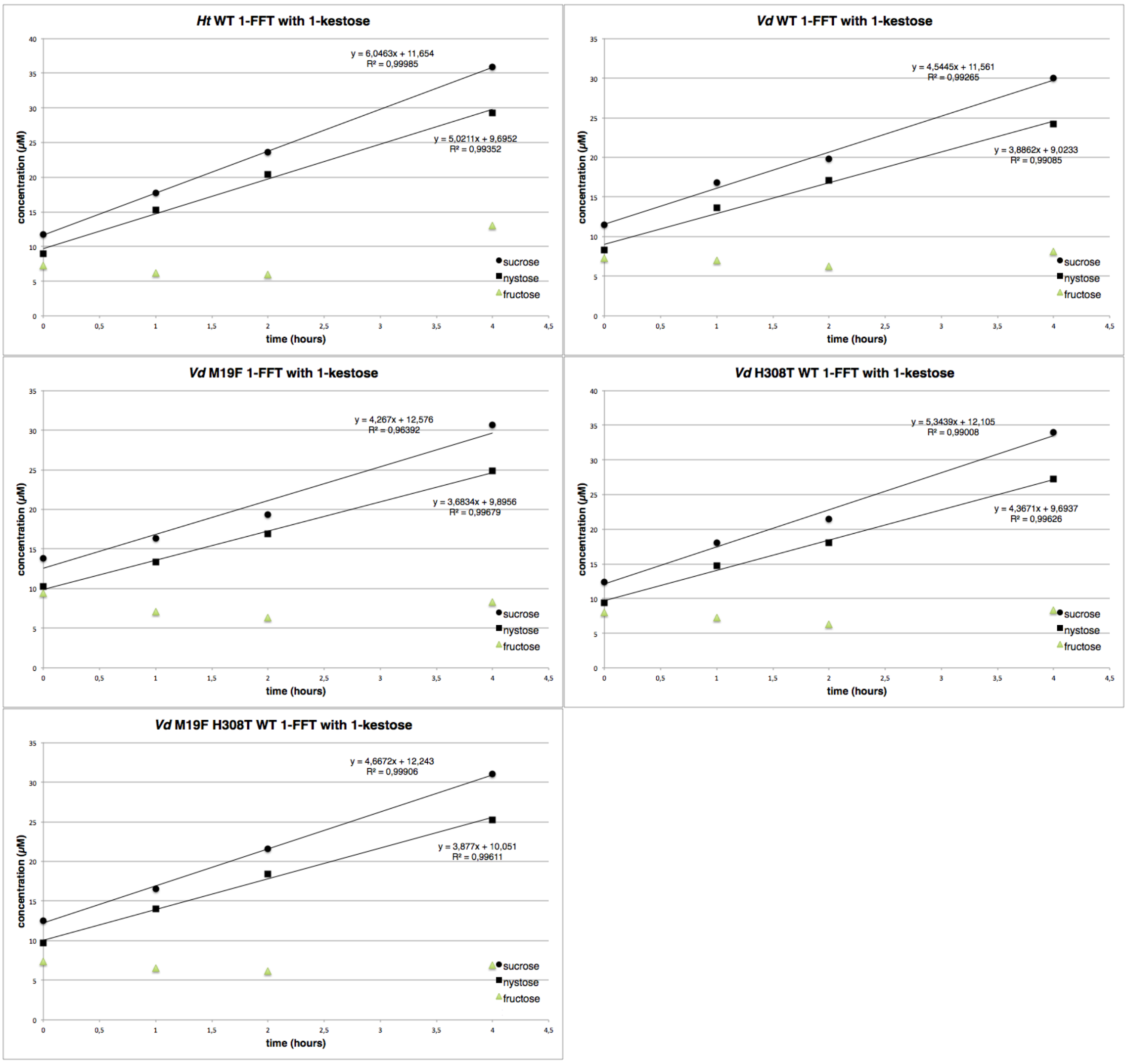

Supplementary Figure 1. Concentration of sucrose and 1,1-kestotetraose produced, in function of time when only 1-kestotriose is used as substrate for the *Ht* and *Vd* 1-FFT wild types and the M19F and H308T single and double mutant of *Vd* 1-FFT, with the linear trend line and its function and R^2 value. Also present in the graphs is the concentration of fructose, in function of time.

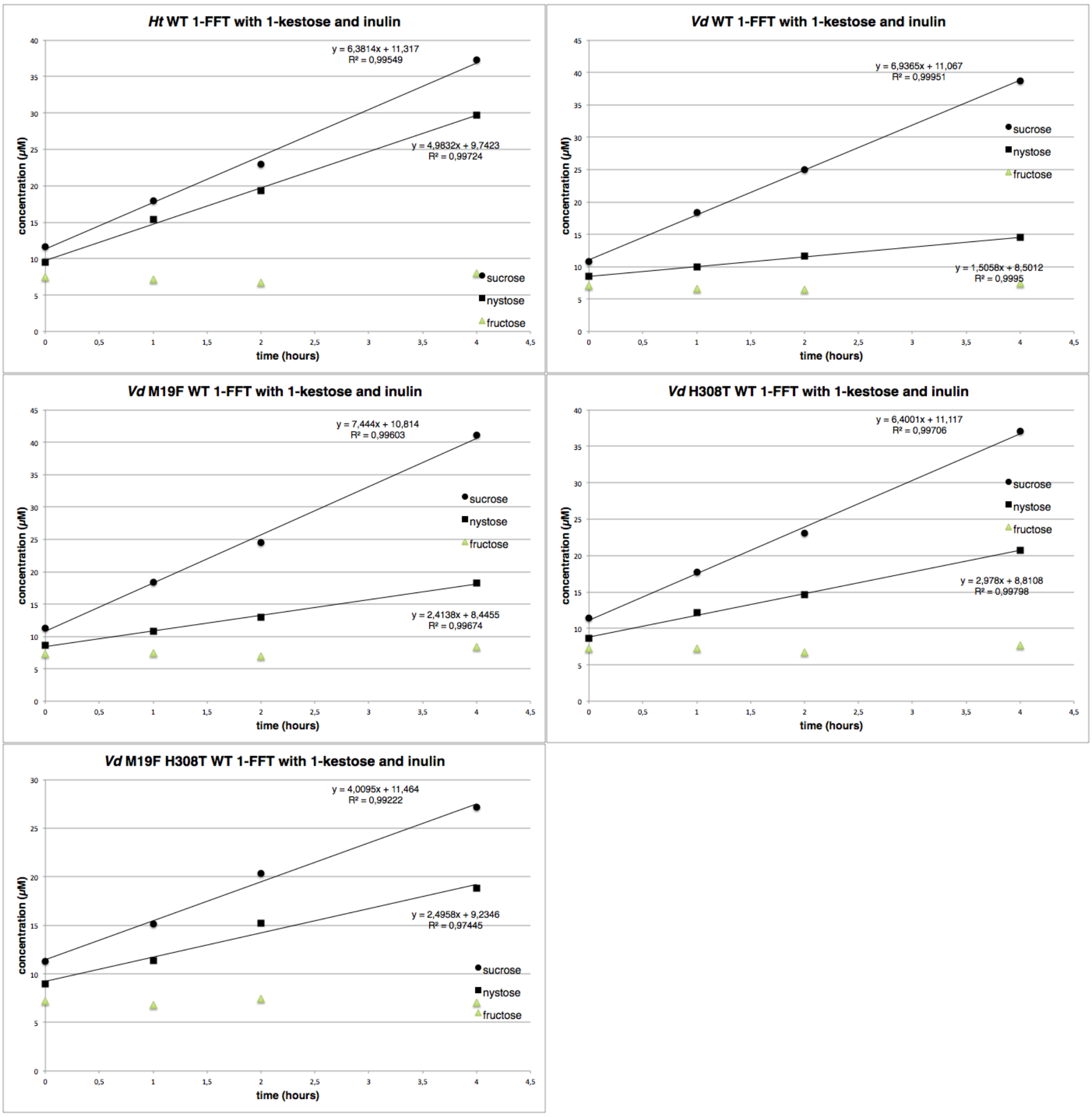

Supplementary Figure 2. Concentration of sucrose and 1,1-kestotetraose produced, in function of time when 1-kestotriose supplemented with inulin is used as substrate for the *Ht* and *Vd* 1-FFT wild types and the M19F and H308T single and double mutant of *Vd* 1-FFT, with the linear trend line and its function and R^2 value. Also present in the graphs is the concentration of fructose, in function of time.

Supplementary Table 1. Mean (with n=3 for the WT and double mutant enzymes and n=2 for the single mutant enzymes) of the ratios of the production rates of sucrose and 1,1-kestotetraose when 1-kestotriose (A) is used as sole substrate or supplemented with inulin (B) with the standard deviation.

enzyme	A	StDev	B	StDev
Ht WT	1.389	0.445	1.401	0.267
Vd WT	1.240	0.238	4.330	0.786
Vd M19F	1.128	0.043	3.096	0.017
Vd H308T	1.119	0.120	2.149	0.001
Vd M19F H308T	1.414	0.396	1.830	0.422
